# Variations in long-term care home resident hospitalizations before and during the COVID-19 pandemic in Ontario

**DOI:** 10.1371/journal.pone.0264240

**Published:** 2022-11-04

**Authors:** Aaron Jones, Fabrice I. Mowbray, Lindsey Falk, Nathan M. Stall, Kevin A. Brown, Kamil Malikov, Sarah L. Malecki, Sharan Lail, Hae Young Jung, Andrew P. Costa, Amol A. Verma, Fahad Razak

**Affiliations:** 1 Department of Health Research Methods, Evidence, and Impact, McMaster University, Hamilton, ON, Canada; 2 Michael G. DeGroote School of Medicine, Hamilton, Ontario, Canada; 3 Division of General Internal Medicine and Geriatrics, University Health Network and Sinai Health System, Toronto, Ontario, Canada; 4 Department of Medicine, University of Toronto, Toronto, Ontario, Canada; 5 Women’s College Hospital Research Institute, Toronto, Ontario, Canada; 6 Public Health Ontario, Toronto, Ontario, Canada; 7 Dalla Lana School of Public Health, University of Toronto, Toronto, Ontario, Canada; 8 Health Data Science Branch, Capacity Planning and Analytics Divisions, Ontario Ministry of Health, Toronto, ON, Canada; 9 St. Michael’s Hospital, Unity Health Toronto, Toronto, ON, Canada; 10 Institute of Health Policy, Management, and Evaluation, University of Toronto, Toronto, ON, Canada; University of Florence, ITALY

## Abstract

**Objectives:**

To examine how the COVID-19 pandemic affected the demographic and clinical characteristics, in-hospital care, and outcomes of long-term care residents admitted to general medicine wards for non-COVID-19 reasons.

**Methods:**

We conducted a retrospective cohort study of long-term care residents admitted to general medicine wards, for reasons other than COVID-19, in four hospitals in Toronto, Ontario between January 1, 2018 and December 31, 2020. We used an autoregressive linear model to estimate the change in monthly admission volumes during the pandemic period (March-December 2020) compared to the previous two years, adjusting for any secular trend. We summarized and compared differences in the demographics, comorbidities, interventions, diagnoses, imaging, psychoactive medications, and outcomes of residents before and during the pandemic.

**Results:**

Our study included 2,654 long-term care residents who were hospitalized for non-COVID-19 reasons between January 2018 and December 2020. The crude rate of hospitalizations was 79.3 per month between March-December of 2018–2019 and 56.5 per month between March-December of 2020. The was an adjusted absolute difference of 27.0 (95% CI: 10.0, 43.9) fewer hospital admissions during the pandemic period, corresponding to a relative drop of 34%. Residents admitted during the pandemic period had similar demographics and clinical characteristics but were more likely to be admitted for delirium (pandemic: 7% pre-pandemic: 5%, p = 0.01) and were less likely to be admitted for pneumonia (pandemic: 3% pre-pandemic: 6%, p = 0.004). Residents admitted during the pandemic were more likely to be prescribed antipsychotics (pandemic: 37%, pre-pandemic: 29%, p <0.001) and more likely to die in-hospital (pandemic:14% pre-pandemic: 10%, p = 0.04)

**Conclusions and implications:**

Better integration between long-term care and hospitals systems, including programs to deliver urgent medical care services within long-term care homes, is needed to ensure that long-term care residents maintain equitable access to acute care during current and future public health emergencies.

## Introduction

The COVID-19 pandemic had a dramatic impact on the use of hospital services in Canada, with significant drops in hospital occupancy rates beginning in March of 2020 [1]. The decrease in hospital admissions stemmed from a number of factors, including efforts to proactively preserve bed space for expected COVID-19 surges [2] and a reduction in general care-seeking behavior [3]. While many diverted admissions were likely non-urgent, concerns were raised about the downstream consequences of decreased admissions for life-threatening conditions stroke [4] and cancer [5]. Similar to the broader population, the pandemic led to significant reductions in the volume of admissions of long-term care residents to hospital, despite the intense pressure that long-term care homes faced during the first wave of the pandemic [6].

Existing research on the frequency of long-term care residents admitted to hospital during the COVID-19 pandemic in Canada has been restricted to aggregate analysis at the population level [6, 7]. In particular, there is a lack of research on how the pandemic impacted the patient-level characteristics and care of long-term residents admitted to hospital for non-COVID-19 reasons. The objective of this study was to investigate differences in demographic and clinical characteristics, in-hospital care, and outcomes of long-term care residents admitted to general internal medicine wards for non-COVID-19 reasons, before and during the COVID-19 pandemic.

## Methods

### Design and setting

We conducted a retrospective cohort study within four hospital sites in Toronto, Ontario, Canada.

### Data sources

All data for the study was accessed through the GEMINI research database. GEMINI is a hospital research collaborative [8] that collects and standardizes clinical, demographic, resource use, and outcome data from hospitals in Ontario [9]. We collected demographic, resource use, and outcome data as reported to the Discharge Abstract Database and National Ambulatory Care Reporting System. Clinical, laboratory, radiology, and medication data were extracted from hospital information systems.

### Study population

We included all residents living in long-term care homes admitted to general internal medicine wards for non-COVID-19 reasons at the study sites between January 1, 2018 and December 31, 2020. Participants were restricted to those residents admitted to general medicine wards to enable consistent data capture and avoid selection bias across the three years of the study. We excluded admissions for COVID-19 based on the ICD-10-CA code U07.1 (“COVID-19 diagnosis confirmed by a laboratory test”) [10] and U07.2 (“COVID-19 diagnosed clinically or epidemiologically but lab results inconclusive, unavailable, or not performed”).

### Measures

The pandemic period was defined as March-December 2020 [11]. To avoid seasonal effects, we compared resident characteristics only in those residents admitted between March to December of each year.

#### Demographic and clinical characteristics

We described the residents’ age, sex, and comorbidity profile. We reported the Elixhauser comorbidity index [12] and specific chronic conditions using previously validated definitions [13]. We also calculated the laboratory-based acute physiology score (LAPS) [14] to measure resident’s severity of illness at admission. LAPS is calculated from laboratory data and is a valid prognosticator of in-patient mortality [15]. We extracted the three-digit ICD-10-CA code of most responsible discharge diagnosis from the administrative records and reported the five most commonly documented diagnoses during either period.

#### In-hospital care

We characterized the in-patient care that residents received based on pre-specified categories of clinical management and resource use. For interventions, we reported the proportion of residents that received mechanical ventilation, any modality of dialysis, bronchial or gastrointestinal endoscopy, and red blood cell transfusions. We also reported the use of imaging studies, including plain radiography, computed tomography, magnetic resonance imaging, and ultrasonography. We examined in-hospital use of antidepressants (ATC codes: NO6A, N06AX), antipsychotics (NO5A, NO5AX), and benzodiazepines (NO3AE, NO5BA, NO5CD). We chose these medication classes given evidence of increased use of psychotropic medications in long-term care homes in Ontario across 2020 [16].

#### Outcomes

Outcomes examined included hospital length of stay, admission to an intensive care unit, and in-patient mortality.

### Analysis

We plotted the monthly count of admissions from January 2018 to December 2020. We fit an autoregressive linear model to estimate the change in the average monthly count of admissions during the pandemic period. To achieve this, we included an interaction term between year (2020 vs. 2018–2019) and month category (January-February vs. March-December) while also controlling for a linear secular trend. This allowed us to compare 2020 to the previous years separately for the portions of the year before and during the pandemic.

For residents admitted between March and December, we reported summary statistics for all demographic characteristics, clinical characteristics, outcomes, and in-hospital care measures across the two periods. We examined differences between periods using the Wilcoxon rank-sum test for continuous variables and Fisher’s exact test for categorical variables. All analysis was done using R 3.4.4 [17].

#### Sensitivity analysis

Some patients may have contracted COVID-19 after hospital admission. We sought to identify these patients using post-admission comorbidities diagnoses U07.1 and U07.2 in the Discharge Abstract Database, and examined the effects of excluding these individuals on our findings. We also tabulated descriptive data on patients admitted for COVID-19.

#### Ethics

Ethics approval was obtained from Clinical Trials Ontario (1394) and Mount Sinai Hospital (15-0075-C). Waiver of informed consent was obtained as this was a large retrospective study with minimal risk. All records were full anonymized prior to access.

## Results

Our study included 2,654 long-term care residents who were admitted to one of the study hospitals for non-COVID-19 reasons between January 2018 and December 2020; 2,150 of whom were admitted between March and December. The count of admissions between March and December was 791 (crude rate 79.1 per month) in 2018, 794 (crude rate 79.4 per month) in 2019, and 565 (crude rate 56.5 per month) in 2020. There were 57 COVID-19 positive admissions that were excluded from the pandemic period, 24 (52%) of which were in April 2020. Had the COVID-19 admissions been included, the crude March-December 2020 admission rate would have been 62.2 per month.

### Monthly volume of admissions

**Fig 1:**
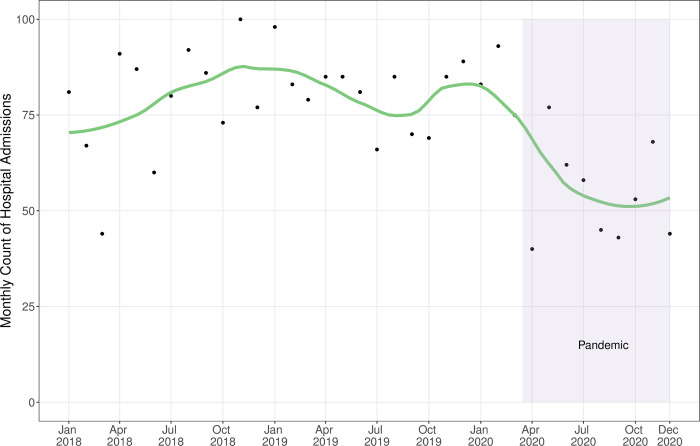
Monthly count of long-term care resident admissions to general internal medicine wards (2018–2020), Toronto, Ontario, pandemic period highlighted.

The times series of monthly hospital admissions indicates a drop in admissions during the pandemic period with little recovery by December 2020 (Fig 1). The monthly relative difference between 2020 and 2018–2019 was greatest in April 2020, in which the monthly admission count was 54.5% lower than the average of the previous two years (Table 1). The autoregressive linear model estimated that the March-December monthly admission count averaged 27.0 (95% CI: 10.0, 43.9) fewer admissions in 2020 compared to 2018–2019 (Table 2), a relative drop of 34%. The difference between 2020 and 2018–2019 for the period January—February was not significant: 1.41 more admissions (95% CI -24.7, 27.5) in 2020.

**Table 1:** Crude absolute and relative differences in average monthly count of hospital admissions, 2020 vs. 2018–2019.

Month	Crude absolute difference in average monthly count of admissions, 2020 vs. 2018–2019	Crude relative difference in average monthly count of admissions, 2020 vs. 2018–2019
January	-6.5	-7.3%
February	18	24.0%
March	13.5	22.0%
April	-48	-54.5%
May	-9	-10.5%
June	-8.5	-12.1%
July	-15	-20.5%
August	-43.5	-49.2%
September	-35	-44.9%
October	-18	-25.4%
November	-24.5	-26.5%
December	-39	-47.0%

**Table 2:** Mean change in monthly count of admissions from autoregressive linear model by month category, 2020 vs. 2018–2019.

Month category	Mean change in monthly count of admissions (95% CI), 2020 vs. 2018–2019	p
January-February	1.41 (-24.7,27.5)	0.91
March-December	-27.0 (-43.9,-10.0)	0.004

### Demographic and clinical characteristics

In the pre-pandemic period, admitted residents had a median age of 84 years and 47% of residents were female. (Table 3). In the pandemic period, the median resident age was 83 (p = 0.84) and 51% of residents were female (p = 0.07). Pandemic residents had similar median LAPS (pre-pandemic: 14, pandemic: 13, p = 0.38) and slightly higher median Elixhauser index scores (pre-pandemic = 6, pandemic = 7, p = <0.001) compared to pre-pandemic residents. Comorbidity profiles between pre-pandemic and pandemic residents were broadly similar.

**Table 3:** Demographic and clinical characteristics, in-hospital care, and outcomes of long-term care residents admitted to general internal medicine wards, March to December, 2018–2020.

	Pre-Pandemic	Pandemic	
	2018–2019	2020	
Characteristic	n = 1585	n = 565	p
**Demographic**			
Age, yrs, (median, q1, q3)	84 (73, 90)	83 (74, 90)	0.84
Sex, F, n (%)	742 (47)	290 (51)	0.07
**Clinical**			
LAPS, (median, q1, q3)^1^	14 (6, 22)	13 (6, 22)	0.38
**Comorbidities**			
Elixhauser index, (median, q1, q3)	6 (0, 11)	7 (3, 12)	<0.001
Dementia	610 (38)	213 (38)	0.76
Congestive Heart Failure	304 (19)	127 (22)	0.1
Chronic obstructive pulmonary disease	224 (14)	72 (13)	0.43
Diabetes	598 (38)	192 (34)	0.12
Hypertension	981 (62)	374 (66)	0.08
Stroke	48 (3)	14 (2)	0.56
**Most Responsible Diagnosis (ICD-10)** ^**2**^			
Aspiration Pneumonia (J69)	169 (11)	58 (10)	0.87
Congestive Heart Failure (I50)	91 (6)	24 (4)	0.19
Pneumonia (J18)	92 (6)	16 (3)	0.004
Sepsis (A41)	95 (6)	41 (7)	0.31
Urinary Tract Infection (N39)	87 (5)	32 (6)	0.91
Delirium (F05)	73 (5)	42 (7)	0.01
**Interventions**			
Mechanical Ventilation, n (%)	16 (1)	8 (1)	0.48
Dialysis, n (%)	47 (3)	13 (2)	0.46
Endoscopy, n (%)	87 (5)	40 (7)	0.18
Blood Transfusion, n (%)	117 (7)	56 (10)	0.07
**Diagnostic Imaging**			
Plain Radiography, n (%)	1413 (89)	506 (90)	0.81
CT, n (%)	969 (61)	387 (68)	0.002
MRI, n (%)	79 (5)	32 (6)	0.5
Ultrasound, n (%)	319 (20)	127 (22)	0.25
**Psychoactive Medications**			
Antipsychotics	465 (29)	210 (37)	<0.001
Antidepressants	586 (37)	225 (40)	0.25
Benzodiazepines	336 (21)	136 (24)	0.16
**Outcomes**			
Length of stay, days, (median, q1, q3)	4.8 (2.7, 8.6)	6.2 (3.4, 10.7)	<0.001
ICU Admission, n (%)	56 (4)	24 (4)	0.44
Death, n (%)	166 (10)	78 (14)	0.04

1. Laboratory acute physiology scale

2. Top 5 most frequent three-digit most responsible diagnoses

The five most frequent most responsible diagnoses among pre-pandemic residents were Congestive Heart Failure (I50), Aspiration Pneumonia (J69), Pneumonia (J18), Urinary Tract Infection (N39), and Sepsis (A41). Among pandemic residents, Delirium (F05) was also a top five most responsible diagnosis, displacing Pneumonia (J18).

### In-hospital care

Intervention rates were similar across both groups. Nearly all residents in both groups received radiographs (pre-pandemic: 89%, pandemic: 90%, p = 0.81). Pandemic residents were more likely to receive computed tomography, although the difference was small (pre-pandemic: 61%, pandemic 68%, p = 0.002). Use of magnetic resonance imaging and ultrasonography where similar between the groups. Antipsychotic medications were more frequently dispensed in-hospital during the pandemic period (37%) compared to the pre-pandemic period (29%, p = <0.001). Antidepressants (pre-pandemic: 37%, pandemic = 40%, p = 0.25) and benzodiazepines (pre-pandemic:21%, pandemic = 24%, p = 0.16) were not significantly different.

### Outcomes

Hospital length of stay was longer for pandemic residents, who had a median stay of 6.2 days compared to 4.8 days for pre-pandemic residents (p <0.001). While the rates of ICU admission were similar, pandemic residents were more likely to die in hospital (14%) compared to 10% for pre-pandemic residents (p = 0.04).

### Sensitivity analysis

Only three residents were identified with post-admit COVID-19. Their exclusion did not meaningfully alter any results. Descriptive data on the COVID-19 admissions excluded from the main analysis is provided in S1 Table. Compared to non-COVID-19 admissions, residents admitted for COVID-19 were slightly younger (median age 81), had a lower LAPS (median 7), and were more likely to die in-hospital (49%).

## Discussion

We found that non-COVID-19 related admissions of long-term care residents to hospital dropped by over one-third during the pandemic, compared to the previous two years, and did not exhibit signs of recovery through the end of 2020. Residents admitted during the pandemic period for non-COVID-19 reasons were similar in most demographic and clinical characteristics to those admitted during the pre-pandemic period, but had longer hospital stays and were more likely to die in-hospital. Pandemic residents were less likely to be admitted for pneumonia but more likely to be admitted for delirium. Residents received similar care in-hospital between the periods, but pandemic residents were more likely to be prescribed antipsychotic medications.

The drop in hospital admissions starting in April of 2020 mirrors the drops in health care use more broadly in the system at that time [1]. Our study adds to the literature by demonstrating that the volume of long-term care admissions continued below pre-pandemic levels through the end of 2020. This marks long-term care admissions to hospital as an outlier when compared to many other health care services, most of which dipped in the Spring of 2020, but rebounded to pre-pandemic levels by the end of the summer [18, 19]. For example, hospital admissions among the general older adult population in Ontario experienced a peak drop of 46% in April 2020, but recovered to normal levels by the end of the year [20]. In the present study, the peak drop in admissions observed in April 2020 was similar (55%), but hospital admissions remained below normal levels throughout 2020.

The care provided to residents in long-term care homes was inevitably affected by the COVID-19 pandemic. Notably, visits by essential caregivers were heavily restricted and a pre-existing staffing shortage escalated into a staffing crisis [11, 21]. These factors may have reduced the number of hospital transfers due to lack of caregiver advocacy and availability of staff to initiate and coordinate transfers. Additional on-site medical care was provided to a limited number of homes in a time-restricted manner. The Canadian Armed Forces provided support to 7 of 623 homes in Ontario [22]. Other homes were paired with acute care facilities on a temporary emergency basis, partnerships that likely improved access to acute hospital care [23].

Mortality rates for long-term care residents were significantly higher than normal between March-May and November-December of 2020 [24] and new placements into long-term care homes were markedly below normal levels from March to December [25]. This may have reduced the long-term care population in Ontario and been partially responsible for the drop in hospital admission observed in this study. While the exact count of long-term care residents is not available, publicly reported figures on the number of regular clinical assessments performed in long-term care homes were 10–15% lower during the pandemic period in 2020 compared to the same time period in 2019 [25]. If this figure corresponds to a population decline, then approximately 30–45% of the drop in hospital admissions we observed could be attributable to a reduction in population by the end of the 2020. Notably however, the change in population would not impact the initial drop in hospital admissions in April 2020 and would truncate but not eliminate the enduring reduction in hospital admissions.

The demographic and clinical characteristics of pre-pandemic and pandemic residents admitted to hospitals were similar in most respects. Pneumonia was a considerably less frequent reason for hospitalization during the pandemic period, which is likely related to lower levels of circulating respiratory viruses resulting from public health restrictions to contain the COVID-19 pandemic [26, 27]. Residents spent nearly a day and a half longer in hospital during the pandemic period. This could reflect higher care needs or delays returning residents to long-term care homes during the pandemic due to Ontario policies requiring a negative COVID test. Residents were also significantly more likely to die during the pandemic period, possibly explained by the observed differences in case-mix, patient comorbidity, or by unmeasured factors. This higher in-hospital mortality rate is concerning as most persons prefer to die with family in a home setting, which was not possible for many hospitalized patients, particularly given restrictions on visitors to hospital during the pandemic.

Care in the hospital was similar between pandemic and pre-pandemic residents with the exception of higher use of antipsychotic medications during the pandemic period. Increased use of psychotropic medications during the pandemic has been previously noted with concern within long-term care homes in Ontario [16, 28] and our study extends this finding to the hospital sector. Among the likely drivers of this increase in antipsychotics is restrictive hospital visitation policies which increase social isolation and reduce opportunities for caregiver advocacy [29, 30]. We also observed that admissions for delirium were more common during the pandemic period, which may be another consequence of increased social isolation [31]. Restrictive visitation and socialization policies both in long-term care and inpatient settings during the first wave of COVID-19 resulted in marked increases in social isolation [32]. Mounting evidence has linked increased social isolation during the pandemic to adverse effects on the mental and physical health of older adults [33, 34]. In response, many health systems and governments have developed policies and recommendations to counter social isolation in older adults [35, 36].

The enduring drop in hospitals admissions raises questions regarding the on-going access of long-term care home residents to acute care services. While not all hospital transfers may be necessary, our data do not suggest that residents transferred during the pandemic were of higher acuity, as might be expected if only the more urgent transfers were taking place. Concerns have been raised in the broader population about “missing” hospital admissions and emergency department visits during the early portions of the pandemic for serious conditions necessitating hospital-based care [37, 38]. Our findings suggest that the same concerns are warranted for residents of long-term care homes, in which the situation may be compounded by restrictions on visits by caregivers [39]. Programs that improve the delivery of urgent medical care services within long-term care homes without hospital admissions may represent an opportunity to enhance access to care for long-term care residents [40], but require careful evaluation to ensure care needs are being met.

### Limitations

Our study only included four hospitals in Toronto, Ontario which limited our study size and may impact generalizability. Additionally, we only could consider residents admitted to general medicine wards rather than surgical or other types of wards due to data availability. For our classification of admissions as COVID-19 related, we did not have access to PCR testing results but relied on the diagnoses provided in the hospital record. It is possible that more patients acquired COVID-19 in-hospital than we were able to identify using our data sources. Finally, we were unable to accurately measures changes to the long-term care population in the surrounding homes to analytically adjust for its influence on hospitalization rates.

## Conclusions

The COVID-19 pandemic resulted in an enduring drop in the volume of long-term care residents admitted to hospital. The characteristics of non-COVID-19 residents admitted before and during pandemic were similar, although those admitted during the pandemic were more likely to die in hospital, had differences in case mix, and had higher rates of antipsychotic use. Preparing Ontario’s long-term care system to weather the remainder of the COVID-19 pandemic and to plan for future public health emergencies will require better integration across the hospitals and long-term care systems, to ensure that frail and vulnerable long-term care residents do not experience interruptions in access to acute care.

## Supporting information

S1 Table Demographic and clinical characteristics, outcomes, and in-hospital care of long-term care residents admitted to general internal medicine wards for COVID-19, March to December 2020. (DOCX)
