# Supplementary material for: Variations in long-term care home resident hospitalizations before and during the COVID-19 pandemic in Ontario
Source: PLoS One. 2022 Nov 4;17(11):e0264240. doi: 10.1371/journal.pone.0264240 (PMC9635742; doi:10.1371/journal.pone.0264240)
Supplement: S1 Table — (DOCX) [file pone.0264240.s001.docx]

**S1 Table. Demographic and clinical characteristics, outcomes, and in-hospital care of long-term care residents admitted to general internal medicine wards for COVID-19, March to December 2020**

|  | **COVID-19 Admissions** |
| --- | --- |
| **Characteristic** | **n = 57** |
| **Demographic** |  |
| Age, yrs, (median, q1, q3) | 81 (76, 89) |
| Sex, F, n (%) | 29 (51) |
|  |  |
| **Clinical** |  |
| LAPS, (median, q1, q3)^1^ | 7 (5, 15) |
|  |  |
| **Comorbidities** |  |
| Elixhauser index, (median, q1, q3) | 5 (0, 10) |
| Dementia | 25 (44) |
| Congestive Heart Failure | 12 (21) |
| Chronic obstructive pulmonary disease | 10 (18) |
| Diabetes | 24 (42) |
| Hypertension | 32 (56) |
| Stroke | 0 (0) |
|  |  |
| **Most Responsible Diagnosis (ICD-10)** |  |
| Aspiration Pneumonia (J69) | 0 (0) |
| Congestive Heart Failure (I50) | 0 (0) |
| Pneumonia (J18) | 0 (0) |
| Sepsis (A41) | 0 (0) |
| Urinary Tract Infection (N39) | 0 (0) |
| Delirium (F05) | 0 (0) |
|  |  |
| **Interventions** |  |
| Mechanical Ventilation, n (%) | <6 |
| Dialysis, n (%) | <6 |
| Endoscopy, n (%) | <6 |
| Blood Transfusion, n (%) | <6 |
|  |  |
| **Diagnostic Imaging** |  |
| Plain Radiography, n (%) | 55 (96) |
| CT, n (%) | 32 (56) |
| MRI, n (%) | <6 |
| Ultrasound, n (%) | 9 (16) |
|  |  |
| **Psychoactive Medications** |  |
| Antipsychotics | 24 (42) |
| Antidepressants | 23 (40) |
| Benzodiazepines | 18 (32) |
|  |  |
| **Outcomes** |  |
| Length of stay, days, (median, q1, q3) | 10.8 (4.0, 23.8) |
| ICU Admission, n (%) | 6 (11) |
| Death, n (%) | 28 (49) |
| 1. Laboratory acute physiology scale |  |
